# Treadmill Exercise During Cerebral Hypoperfusion Has Only Limited Effects on Cognitive Function in Middle-Aged Subcortical Ischemic Vascular Dementia Mice

**DOI:** 10.3389/fnagi.2021.756537

**Published:** 2021-12-21

**Authors:** Ryo Ohtomo, Hidehiro Ishikawa, Keita Kinoshita, Kelly K. Chung, Gen Hamanaka, Gaku Ohtomo, Hajime Takase, Christiane D. Wrann, Hiroshi Katsuki, Atsushi Iwata, Josephine Lok, Eng H. Lo, Ken Arai

**Affiliations:** ^1^Neuroprotection Research Laboratory, Departments of Radiology and Neurology, Massachusetts General Hospital and Harvard Medical School, Charlestown, MA, United States; ^2^Department of Neurology, Graduate School of Medicine, The University of Tokyo, Tokyo, Japan; ^3^Department of Chemico-Pharmacological Sciences, Graduate School of Pharmaceutical Sciences, Kumamoto University, Kumamoto, Japan; ^4^Cardiovascular Research Center, Department of Medicine, Massachusetts General Hospital and Harvard Medical School, Charlestown, MA, United States; ^5^McCance Center for Brain Health, Massachusetts General Hospital, Boston, MA, United States; ^6^Department of Neurology, Tokyo Metropolitan Geriatric Medical Center Hospital, Tokyo, Japan; ^7^Pediatric Critical Care Medicine, Department of Pediatrics, Massachusetts General Hospital, Boston, MA, United States

**Keywords:** aging, behavior, cognitive function, mouse, subcortical ischemic vascular dementia, treadmill exercise

## Abstract

Clinical and basic research suggests that exercise is a safe behavioral intervention and is effective for improving cognitive function in cerebrovascular diseases, including subcortical ischemic vascular dementia (SIVD). However, most of the basic research uses young animals to assess the effects of exercise, although SIVD is an age-related disease. In this study, therefore, we used middle-aged mice to examine how treadmill exercise changes the cognitive function of SIVD mice. As a mouse model of SIVD, prolonged cerebral hypoperfusion was induced in 8-month-old male C57BL/6J mice by bilateral common carotid artery stenosis. A week later, the mice were randomly divided into two groups: a group that received 6-week treadmill exercise and a sedentary group for observation. After subjecting the mice to multiple behavioral tests (Y-maze, novel object recognition, and Morris water maze tests), the treadmill exercise training was shown to only be effective in ameliorating cognitive decline in the Y-maze test. We previously demonstrated that the same regimen of treadmill exercise was effective in young hypoperfused-SIVD mice for all three cognitive tests. Therefore, our study may indicate that treadmill exercise during cerebral hypoperfusion has only limited effects on cognitive function in aging populations.

## Introduction

Physical activity helps to promote and maintain brain health, including memory retention and cognitive performance. Research has shown that increased physical activity both prevents and ameliorates multiple brain diseases, including subcortical ischemic vascular dementia (SIVD). SIVD is the most common form of vascular cognitive impairment and dementia (VCID) (Erkinjuntti et al., 2000a,b). SIVD patients typically suffer from peri-ventricular white matter degeneration that leads to stepwise development of neurological deficits and loss of executive function, such as difficulties with working memory (Roman et al., 2002; Roh and Lee, 2014; Prins and Scheltens, 2015; Wallin et al., 2018). Although SIVD is expected to become more prevalent as the population ages, to date, there are no clinically effective drugs.

There is an emerging body of evidence suggesting that patients with mild VCID perform better at cognitive functioning tests after aerobic exercise (Liu-Ambrose et al., 2016). However, there is a lack of basic data in experimental models that supports the efficacy of exercise for preventing SIVD/VCID progression. Recently, we reported that treadmill exercise improved cognitive function and increased the number of oligodendrocyte precursor cells (OPCs) in the white matter of hypoperfused-SIVD mice (Ohtomo et al., 2020). Nevertheless, an important question remains; although aging is a major risk factor for SIVD, and the majority of SIVD patients are elderly, no reports to date have examined the efficacy of exercise in aged SIVD mice. Therefore, in this study, we asked whether treadmill exercise alleviates cognitive decline by cerebral hypoperfusion in 8-months old mice.

## Materials and Methods

### Overall Experimental Design

**FIGURE 1:**
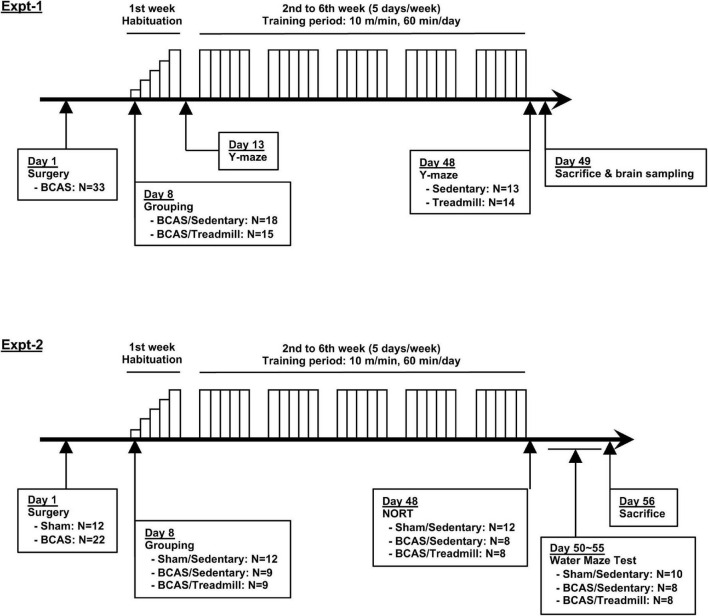
Schematic overview of this study. Two experiments were conducted to examine the effects of treadmill exercise on the cognitive function of middle-aged hypoperfused SIVD-mice. BCAS mice were separated into 2 groups (Sedentary vs. Treadmill) 7 days after the surgery. Following a week-long habituation period (from day 8 to day 12), mice in the treadmill group were placed on a treadmill, on which they were obligated to run for 60 min/day at a maximum speed of 10 m/min on weekdays for 5 weeks. Y-maze was conducted 2 times, on day 13 and day 48 (Experiment 1). NORT was conducted 2 times, on day 48 (Experiment 2). The Morris water maze was conducted between days 50–55 (Experiment 2).

In this mouse model of SIVD, cognitive function (working memory, cognitive memory, and special learning) is known to decline in 4 weeks, presumably due to rarefaction of the white matter (Shibata et al., 2004, 2007; Ihara and Tomimoto, 2011; Yang et al., 2016; Ohtomo et al., 2020; Takase et al., 2021). Our intervention was a 6-week treadmill training which, according to our hypothesis, would slow cognitive decline and ameliorate the deterioration in performance over time. We hypothesized this because this treadmill protocol was shown to be effective in multiple CNS disease models, including young hypoperfused-SIVD mice (Ohtomo et al., 2020; Kinoshita et al., 2021). In this study, we conducted two experiments; Experiment 1 was for Y-maze test, and Experiment 2 was for novel object recognition test (NORT) and Morris water maze test (Figure 1). In both experiments, after the Sham or bilateral common carotid artery stenosis (BCAS) surgery on day 1, treadmill training occurred from day 8 to day 47 in the training group. Mice in the sedentary group were placed on the treadmill for an equivalent amount of time, but without running. In addition to the memory assessment, we also conducted immunohistological and biochemical analyses using mouse brain sections prepared from the mice in Experiment 1. For Experiment 1, 33 mice were used, and 6 mice (5 mice of the BCAS/Sedentary group and 1 mouse of the BCAS/Treadmill group) were excluded due to death or unhealthy conditions. For Experiment 2, 34 mice were used, and 6 mice were excluded due to dead or unhealthy conditions.

### Prolonged Cerebral Hypoperfusion Model (BCAS Mice)

All performed experiments followed an institutionally approved protocol in accordance with the National Institutes of Health Guide for the Care and Use of Laboratory Animals and the law for the humane treatment and management of animals. Eight-month-old male C57BL/6J mice (Jackson Laboratory, United States) were housed in a specific pathogen-free conditioned 12-hour light/dark cycle room with free access to food and water throughout the experiment. After a week-long habituation period in our animal facility, mice were randomly selected to have a microcoil (0.18 mm diameter; Samini, Japan) applied to bilateral common carotid arteries for the induction of chronic cerebral hypoperfusion. The surgical procedure was performed as previously described (Shibata et al., 2004; Ohtomo et al., 2020). A control group received sham operation (after exposing bilateral common carotid arteries, the cervical incision was closed without coil application).

### Treadmill Training Protocol

Sham or BCAS-operated mice were randomly divided into a sedentary group and a training group. The training group was forced to run on a treadmill device (Exter 3/6 Treadmill, Columbus Instruments, United States) for 6 weeks (in the early afternoon, 5 days a week on weekdays). Running speed suitable for exercise was determined from our previous studies that demonstrated protective effects against brain injury (Ohtomo et al., 2020; Kinoshita et al., 2021), beginning with a speed of 2 m/min, with an increase of 2 m/min every 2 min, until a maximum speed of 10 m/min was reached. The length of the total exercise was initially set at 20 min and increased daily by 10 min up to 60 min for familiarization, as shown in Figure 1. The sedentary group was placed on the device without running for the same amount of time as the training group. Mice were always placed in the same lane throughout the experiment.

### Evaluation of the Muscle Fibers of Gastrocnemius Muscles

On day 49, the right gastrocnemius muscle was excised from randomly selected BCAS/Sedentary (*N* = 10) or BCAS/Treadmill (*N* = 10) mice from Experiment 1, under anesthesia with isoflurane before transcardial perfusion. Muscles perpendicularly embedded to Cryomold (Sakura Finetek USA, United States) with Tissue-Tek^®^ (Sakura Finetek USA) were quickly frozen by liquid nitrogen and kept at −80°C until use. Five samples were randomly selected from each study group, and 10 μm-thick frozen sections were made using cryostat CM1520 (Leica, Germany). Sections were then fixed with −20°C methanol for 10 min and stained with Hematoxylin 2 (Thermo Fisher Scientific) and Eosin-Y (Thermo Fisher Scientific) according to the manufacturer’s instructions. Stained sections were observed with ECLIPSE Ti-S (Nikon, Japan) and scanned with Retiga™ 2000R Fast 1394 Digital Camera (QImaging, Canada). Then, a cross-sectional area of 50 muscle fibers per mouse was measured by using ImageJ^1^. To compare the area distribution of the muscle fibers between the groups, a histogram was drawn using PRISM^®^ 7 (GraphPad Software, United States).

### Y-Maze

After the first and sixth week of exercise (day 13 and day 48), mice were tested for spontaneous alternation behavior with Y-maze between 6 AM and 9 AM. Our approach in conducting the Y-maze test multiple times in the same mouse was justified by the papers that the retention memory within the Y-maze task may not last longer than a few hours (Dellu et al., 2000; Fu et al., 2017). Each mouse was placed in the arm of symmetrical Y-maze apparatus to freely explore the maze for 8 min. The task was videotaped, and the sequence and the total number of arm entries were manually recorded later in a blinded manner. An arm entry was confirmed when bilateral hind paws were placed inside the arm. Arms were washed with 70% Ethanol between each test. Percentage of alternation was calculated as follows: number of triads containing entries into 3 different arms/(total number of arms entered −2) × 100.

### NORT

Mice were tested for recognition memory by NORT between 8 AM and 1 PM on day 48. At first, the mice were habituated in an empty cage for 10 min before training. During training, they experienced 5-min exposure to 2 identical plastic blocks 2 times. After 30 min, they were then presented with 2 different plastic blocks (one of the original blocks replaced by a new block) for 5 min. The mouse behavior was videotaped and manually assessed in a blinded manner. Object recognition was scored by the total time spent either sniffing or touching the object. The performance of recognition memory was evaluated by the ratio of the time spent on the new object to the total time spent on 2 objects (Ohtomo et al., 2020). Five mice were excluded from analysis due to lack of interest toward the objects (e.g., those mice did not sniff or touch the 2 objects).

### Morris Water Maze Test

On days 50–55, mice were tested with Morris water maze to evaluate spatial learning and memory as previously described (Ohtomo et al., 2020). In brief, mice had to find a hidden (2 cm under the water surface) escape platform (diameter: 11.5 cm) which was placed in the northeastern quadrant of a circular pool (diameter: 110 cm) filled with opacified water kept at 25°C. In the acquisition phase, mice performed 4 trials per day from a random starting position for 5 consecutive days, with a maximum trial duration of 60 s. To test spatial memory, the platform was removed, and 60-second probe trials were performed on day 54 (4 h after the last acquisition test) and day 55 (4 h after the last acquisition test). Escape latency to find the hidden platform and number of entries into quadrants were examined using ANY-maze video tracking software (Stoelting Co., United States). Due to passive floating behavior in the water, 2 mice were excluded from analysis (one from the BCAS/Sedentary, and the other from the BCAS/Treadmill group). For the Sham/Sedentary group, we randomly picked 10 mice out of 12 Sham/Sedentary mice for the Morris water maze test due to time scheduling issues.

### Western Blot Analysis

Mouse brains were removed following transcardial perfusion with 40 mL PBS (0.1 M), quickly frozen in dry ice, and kept at -80°C until use. Frozen brains were thawed to a semi-frozen state, and three 2 mm-thick coronal sections were obtained from the forebrain 1–7 mm anterior to the confluence of sinuses using the mouse brain matrix slicing tool. The brain sections were placed in ice-cold PBS, and the meninges were removed, after which the corpus callosum and cerebral cortex were carefully removed under a microscope with a disposable micro knife (Fine Science Tools, Canada). Four times the amount of PBS with protease inhibitor cocktail (Cytoskeleton, United States) was promptly added to the samples and dismembraned by sonic dismembrator (ThermoFisher Scientific, United States) on ice. After adding Triton X-100 (Sigma-Aldrich, United States) to a final concentration of 1%, samples were frozen at −80°C and thawed on ice. Then, they were centrifuged at 10000 × *g* for 15 min at 4°C to remove cellular debris. Protein concentration was determined by the BCA assay (Thermo Fisher, United States). Collected samples were heated with equal amounts of LDS sample buffer (ThermoFisher Scientific) and sample reducing agent (ThermoFisher Scientific) at 70°C for 10 min. Each sample was equally (30 μg) loaded onto 4–12% Bis-Tris gels (ThermoFisher Scientific) for electrophoresis, followed by transfer to a nitrocellulose membrane (Thermo Fisher Scientific). Membranes were blocked in 5% skim milk (LabScientific, United States) and incubated overnight at 4°C with primary antibodies against PDGFRα (1:500, Sigma-Aldrich, United States), MBP (1:1000, Thermo Fisher Scientific), and β-actin (1:10000, Sigma-Aldrich). Then, membranes were processed with peroxidase-conjugated secondary antibodies [1:1000 for anti-rabbit antibody (GE Healthcare, United States), and 1:2000 for anti-mouse antibody (GE Healthcare, United States)] and visualized by enhanced chemiluminescence (Thermo Fisher Scientific). Visualized bands were semi-quantified with ImageJ.

### Immunohistochemistry

Five samples were randomly selected from each study group, and 20 μm-thick coronal sections (corresponding to the area 0–1 mm anterior to bregma) were prepared for immunostaining using cryostat CM1520. Frozen sections were then fixed with −20°C methanol for 10 min. After being washed with PBS containing 0.1% Triton X-100 for 5 min 3 times, they were incubated in PBS/3% BSA solution for 1 h at room temperature. Then, sections were incubated in PBS/3% BSA solution containing primary antibodies anti-PDGFRα (1:100, R&D systems, United States) or anti-nestin (1:100, Abcam, United States) at 4°C overnight. After being washed with PBS 3 times, they were incubated with secondary antibodies (1:1000, Jackson Immunoresearch Laboratories) for 1 h at room temperature. Finally, the sections were washed three times with PBS, and covered with VECTASHIELD^®^ mounting medium with DAPI (Vector Laboratories, United States). Stained sections were observed with ECLIPSE Ti-S and scanned with Retiga™ 2000R Fast 1394 Digital Camera. All brain sections were blinded to the examiner before the evaluation of fluorescence intensity. Immunofluorescence intensity of PDGFRα-positive cells inside the subventricular zone (SVZ) was calculated by ImageJ. Bilateral sides of two coronal sections cut from the area + 0.5 ∼ + 1.0 mm to the bregma were evaluated, and the average of 4 areas was calculated per mouse. The same procedure was conducted for evaluating the fluorescence intensity of nestin-positive cells.

### Statistics

Statistical analysis was performed with R version 3.4.0^2^ and PRISM^®^ 7. For body weight, data were first tested with two-way analysis of variance (ANOVA), followed by *post hoc* Sidak’s multiple comparisons test. For the acquisition phase of the Morris water maze test, data were first tested with two-way ANOVA, followed by *post hoc* Tukey’s multiple comparisons test. Data from the probe test were first analyzed with Brown-Forsythe ANOVA, followed by *post hoc* Dunnett’s T3 multiple comparisons test. NORT data were first analyzed with one-way ANOVA, followed by *post hoc* Tukey’s multiple comparisons test. Mann-Whitney *U* test or Welch’s *t*-test was used for other analyses. All values were expressed as mean ± SD. *P*-values less than 0.05 were considered statistically significant.

## Results

To examine the effects of treadmill exercise on the cognitive function of middle-aged hypoperfused-SIVD mice, we conducted two sets of experiments (Figure 1). Hypoperfused-SIVD mice were prepared by subjecting mice to the BCAS operation. The baseline of cerebral blood flow is lower in aged mice (22 months old), compared to young mice (2–3 months old), thus the decrease of cerebral blood flow by BCAS is less impacted in aged mice (Baik et al., 2021). However, at least in our system, the BCAS operation caused prolonged cerebral blood flow reduction in middle-aged mice (8 months old) (Supplementary Figure 1). In the first set of experiments, we prepared two groups (hypoperfused-SIVD mice without exercise treadmill and hypoperfused-SIVD mice with exercise treadmill) to investigate whether exercise treadmill during cerebral hypoperfusion mitigated cognitive decline (e.g., from day 13 to day 48 after BCAS) using Y-maze test. In addition, after the completion of the Y-maze experiment, gastrocnemius muscles and brains were sampled for biochemical analyses. In the second set of experiments, we prepared three groups (sham mice without exercise treadmill, hypoperfused-SIVD mice without exercise treadmill, and hypoperfused SIVD mice with exercise) to examine whether the cognitive function of hypoperfused-SIVD mice with treadmill exercise would be comparable with that of sham mice without treadmill exercise. For both experiments, male C57BL/6J (8 months old) mice were subjected to the 6-week exercise after sham or BCAS operation. One week was spent on habituation exercise, where running speed began at 2 m/min and was increased by 2 m/min every 2 min until a maximum speed of 10 m/min was reached; the duration of the daily exercise was initially set at 20 min and was increased daily by 10 min up to 60 min. In the following 5 weeks, mice were then subjected to running exercise at a speed of 10 m/min for 60 min/day.

**FIGURE 2:**
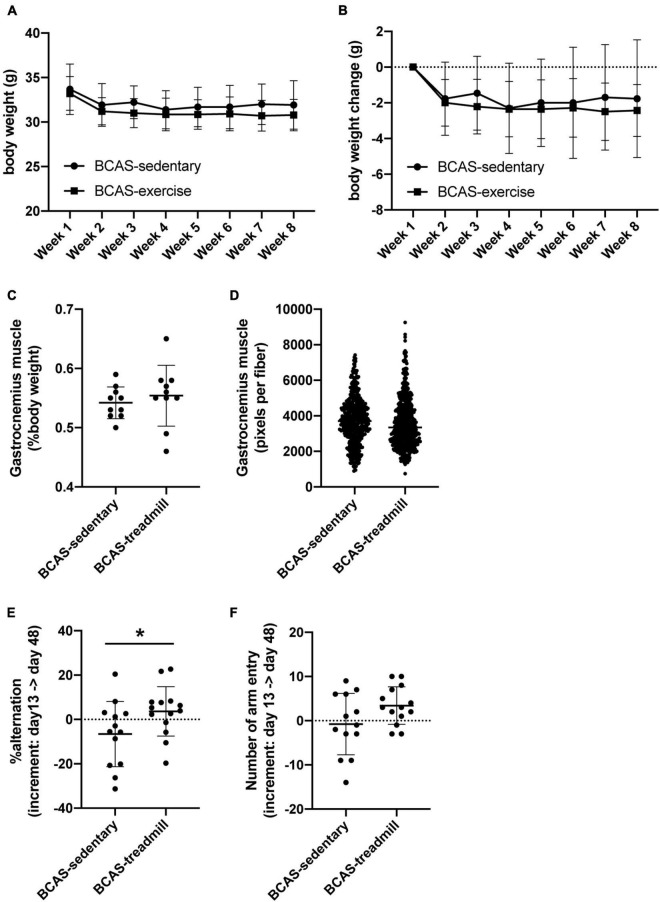
Result of cognitive function test from Experiment 1. **(A,B)** Body weight transition of mice **(A)**, and temporal body weight change of mice **(B)**. There was no significant difference between the 2 groups (BCAS/Sedentary vs. BCAS/Treadmill). Data are expressed as mean ± SD. **(C,D)** Weight of gastrocnemius muscle (percentage of body weight) **(C)** and distribution of the area of muscle fibers **(D)** at day 49. There was no significant difference between the 2 groups (BCAS/Sedentary vs. BCAS/Treadmill). Data are expressed as mean ± SD. **(E,F)** Change in alternation (an index of working memory) in Y-maze test before and after the training period **(E)** and change in the number of arm entries in Y-maze test before and after the training period **(F)**. There was a significant difference in the increment of alteration between the 2 groups (BCAS/Sedentary vs. BCAS/Treadmill) (*P < 0.05, Mann-Whitney *U* test). Data are mean ± SD.

In the first set of experiments, both groups (hypoperfused-SIVD mice without exercise and hypoperfused-SIVD mice with exercise) lost body weight after BCAS operation, and over time during the treadmill exercise training, they did not gain body weight regardless of treadmill exercise (Figures 2A,B). In addition, there was no significant difference in the right gastrocnemius muscles between the two groups (Figure 2C for the percentage of gastrocnemius muscle weight and Figure 2D for the distribution of muscle fiber thickness). However, in the Y-maze tests, while hypoperfused-SIVD mice without treadmill exercise showed a decline in cognitive function from day 13 to day 48 after BCAS operation, hypoperfused-SIVD mice with treadmill exercise did not (Figures 2E,F).

**FIGURE 3:**
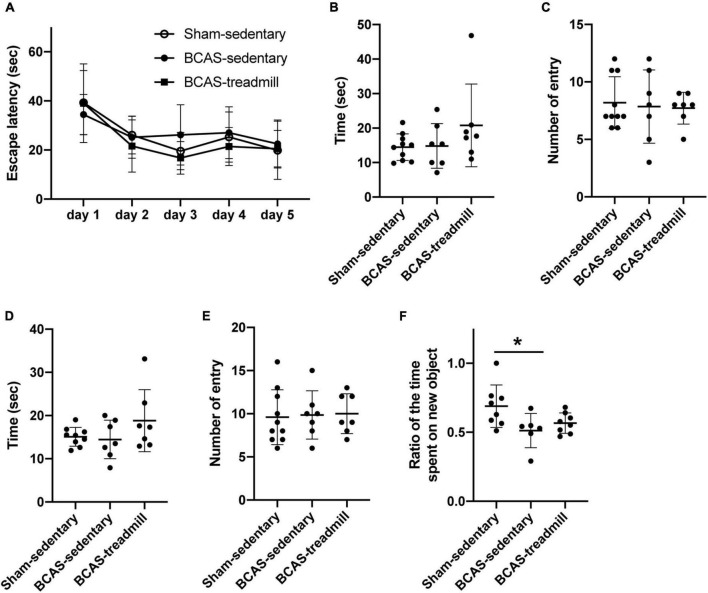
Result of cognitive function test from Experiment 2. **(A)** During the acquisition phase of Morris water maze, no difference was observed in latencies to locate the hidden platform between the 3 groups (Sham/Sedentary vs. BCAS/Sedentary vs. BCAS/Treadmill). Data are mean ± SD. **(B,C)** Result of the probe trial (4 h after the last acquisition session) in the Morris water maze test. There were no significant differences in both **(B)** number of entries into and **(C)** time spent in the quadrant which formerly contained a hidden platform in the acquisition phase. Data are mean ± SD. **(D,E)** Result of the probe trial (24 h after the last acquisition session) in the Morris water maze test. There were no significant differences in both **(D)** number of entries into and **(E)** time spent in the quadrant which formerly contained a hidden platform in the acquisition phase. Data are mean ± SD. **(F)** Compared to the Sham/Sedentary group, the BCAS/Sedentary group showed less preference to the novel object (*p < 0.05, one-way ANOVA followed by *post hoc* Tukey’s test). However, there was no significant difference between Sham/Sedentary and BCAS/Treadmill groups. Data are mean ± SD.

In the second set of experiments, we assessed cognitive function with the Morris water maze test and NORT. In the Morris water maze test, there was no significant difference in the memory acquisition between the three groups (sham mice without exercise vs. hypoperfused-SIVD mice without exercise vs. hypoperfused-SIVD mice with exercise) (Figure 3A). In addition, in the probe tests at 4 and 24 h after the last session of acquisition trial, there were no differences in the memory retention performance between the groups (Figures 3B–D). In NORT, as expected, sham mice without exercise showed a preference for the novel object; however, hypoperfused-SIVD mice without exercise showed no preference between the familiar and novel objects (Figure 3E). On the other hand, there was no significant difference in the preference between sham mice without exercise and hypoperfused-SIVD mice with exercise (Figure 3F).

**FIGURE 4:**
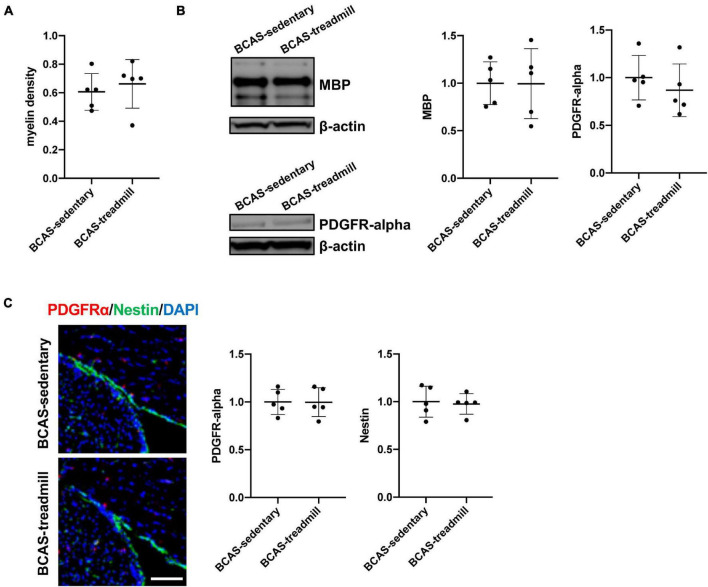
**(A)** Result of myelin density of the corpus callosum, assessed by fluoromyelin staining. Myelin density was calculated based on the intensity of age-matched male C57/BL6J mice (average of 5 mice). There was no significant difference in myelin density between the 2 groups (BCAS/Sedentary vs. BCAS/Treadmill). Data are expressed as mean ± SD. **(B)** Result of MBP and PDGFRα western blot using samples from the corpus callosum region. Data are expressed as mean ± SD. **(C)** Immunohistochemistry showed that there were no significant differences in both the intensity of PDGFRα-positive cells and the intensity of Nestin-positive cells within the SVZ region. Data are expressed as mean ± SD.

Finally, we examined whether white matter pathology would be affected by treadmill exercise, because white matter damage is one of the major characteristics of SIVD. Fluoro-myelin staining showed that there was no difference in myelin density in the corpus callosum between hypoperfused-SIVD mice with or without treadmill exercise (Figure 4A). Western blotting also confirmed that treadmill exercise did not change the myelin density of corpus callosum, assessed by the level of MBP, which is a major component of the myelin sheath (Figure 4B). After white matter damage, compensatory responses would be activated, and OPCs play an important role in increasing the number of myelin-producing oligodendrocytes. However, the number of OPCs was not affected by the treadmill exercise in middle-aged hypoperfused-SIVD mice (Figures 4B,C). In addition, there was no significant difference in the Nestin-positive cells, which can differentiate into both neurons and OPCs in the adult brain after brain injury (Figure 4C). And finally, we checked if the middle-aged SIVD mice had a significant infarction in the cortical area. It is now well accepted that prolonged cerebral hypoperfusion by BCAS does not cause infarcts in young mice, but little is known about this point in middle-aged mice. But we confirmed no ischemic infarction in the cortex by hematoxylin-eosin staining with randomly selected 10 brain samples from the SIVD mice (Supplementary Figure 2).

## Discussion

Our current study demonstrated that in middle-aged mice with prolonged cerebral hypoperfusion, treadmill exercise ameliorates the decline of working memory in the Y-maze test. On the other hand, treadmill exercise was limited in its supportive effects on cognitive function in the Morris water maze test and NORT. Previously, we showed that the same regimen of treadmill exercise suppressed cognitive decline, assessed by the three cognitive function tests (Y-maze, Morris water maze test, and NORT), in young mice with cerebral hypoperfusion (Ohtomo et al., 2020). Our previous study also showed that the treadmill exercise during cerebral hypoperfusion increased the number of OPCs within the SVZ region of young hypoperfused-SIVD mice (Ohtomo et al., 2020), although we did not confirm the positive effects of treadmill exercise on OPC function in this study. Therefore, together with our previous data (Ohtomo et al., 2020), our current study may suggest that the efficacy of treadmill exercise in cognitive function would decrease with age, but treadmill exercise still seems effective in supporting cognitive function even in aging populations. This is consistent with past studies that treadmill exercise supported cognitive function in both young and aged AD mice, but the efficacy was smaller in the aged groups (Choi et al., 2021).

An important aspect of our current study is the finding that treadmill exercise is less effective in middle-aged SIVD model mice. To the best of our knowledge, except for our previous report (Ohtomo et al., 2020), there are only two previous studies that evaluate the effectiveness of exercise in preventing/ameliorating cognitive decline caused by chronic cerebral hypoperfusion (Jiang et al., 2017; Lee et al., 2017). These two studies showed that treadmill exercise is effective in ameliorating cognitive dysfunction using young SIVD model rats. Our findings further add to this existing literature. The efficacy of treadmill exercise during cerebral hypoperfusion in cognitive function might be less effective in aging populations. This may be partly explained by the fact that compensative responses after brain injury, such as neurogenesis and oligodendrogenesis, are dampened by age (Miyamoto et al., 2013; Liang et al., 2016). While the treadmill exercise increased the number of OPCs within the SVZ region in young SIVD mice (Ohtomo et al., 2020), our current study did not confirm this effect in middle-aged mice. In addition, the body weight loss after cerebral hypoperfusion was not recovered by treadmill exercise in middle-aged mice (Figures 2A,B), although the same protocol of treadmill exercise helped young SIVD mice recover their body weight (Ohtomo et al., 2020). In stroke mice, the body weight recovery was associated with an improvement of motor function (Desgeorges et al., 2017; Kinoshita et al., 2021), which may also explain the difference in the efficacy of treadmill exercise between young and middle-aged mice.

Although we have demonstrated that treadmill exercise ameliorates cognitive decline caused by cerebral hypoperfusion to some extent in middle-aged mice, there are some limitations and caveats in this study. First, we used only one protocol of treadmill exercise, which was shown to be effective for young mice (Ohtomo et al., 2020; Kinoshita et al., 2021). In general, in aged populations, the capability of movement and the total volume of muscle is lower compared to younger populations, so the exercise protocol for young mice may not be suitable for middle-aged mice. The optimization of the conditions of treadmill exercise for middle-aged (or even aged) mice would be necessary for future studies to pursue exercise as a therapeutic option for SIVD and other CNS disease patients. Second, because we focused on the effect of treadmill exercise on middle-aged SIVD mice, our current study did not include a sham-exercise group in our experiments. However, considering that treadmill exercise improves cognitive function in older mice (Maejima et al., 2018), further studies would be needed to answer the question of whether the effect of treadmill exercise is ineffective in middle-aged mice or only in middle-aged SIVD mice in our system. Careful comparison of the effect of treadmill exercise between these two groups may also help us to find a therapeutic target for SIVD. Third, our current study does not address the potential mechanism behind the effects of exercise on cognitive function. In young mice, the activation of compensatory responses, such as an increase of OPC number within the SVZ region, may partially contribute (Ohtomo et al., 2020); however, no OPC activation was observed in middle-aged mice. The hippocampal region plays an important role in cognitive function, including spatial learning and memory, and our pilot study indicated that in the SGZ region of hippocampus, treadmill exercise may increase the number of stem cells (data not shown). Because accumulating evidence now suggests the close relationship between exercise and the neuronal responses in the hippocampus (Moon et al., 2016; Liu and Nusslock, 2018; Lourenco et al., 2019; Baik et al., 2021), it would be helpful to examine the cellular and molecular signaling in the hippocampus after treadmill exercise in middle-aged SIVD mice in future studies. The final caveat of our study is that we used only male mice for our experiment. Since the sex-difference may alter the dynamics of systemic humoral factors as mentioned in the published literature (Herson et al., 2013; McMullan et al., 2016; Stanford et al., 2017), it will be important to determine if our results can be replicated in middle-aged female mice.

In conclusion, we have demonstrated that treadmill exercise has some effect in reducing cognitive decline in middle-aged mice with prolonged cerebral hypoperfusion that mimics the pathophysiology of SIVD. Exercise is a safe behavioral intervention and has the potential to be a non-pharmacological therapy for several CNS diseases, including SIVD. Because polypharmacy among elderly patients has become a serious social issue around the world (Fried et al., 2014), future studies are warranted to pursue the therapeutic option of exercise as a non-pharmacological approach to decrease the cognitive decline in SIVD or other dementia patients.

## Data Availability Statement

The original contributions presented in the study are included in the article/Supplementary Material, further inquiries can be directed to the corresponding author/s.

## Ethics Statement

The animal study was reviewed and approved by the Massachusetts General Hospital Institutional Animal Care and Use Committee.

## Author Contributions

RO, HI, KK, GH, and HT: collection of data. RO, KK, HI, and GO: data analysis. RO, KC, JL, EL, and KA: manuscript writing. RO, KC, GO, CW, HK, AI, JL, EL, and KA: interpretation. RO, GO, EL, and KA: conception and design. EL and KA: funding acquisition. All authors: final approval of manuscript. All authors contributed to the article and approved the submitted version.

## Conflict of Interest

The authors declare that the research was conducted in the absence of any commercial or financial relationships that could be construed as a potential conflict of interest.

## Publisher’s Note

All claims expressed in this article are solely those of the authors and do not necessarily represent those of their affiliated organizations, or those of the publisher, the editors and the reviewers. Any product that may be evaluated in this article, or claim that may be made by its manufacturer, is not guaranteed or endorsed by the publisher.

## Footnotes

1
https://imagej.nih.gov/

2
https://www.r-project.org/
