# Supplementary material for: Treadmill Exercise During Cerebral Hypoperfusion Has Only Limited Effects on Cognitive Function in Middle-Aged Subcortical Ischemic Vascular Dementia Mice
Source: Front Aging Neurosci. 2021 Dec 21;13:756537. doi: 10.3389/fnagi.2021.756537 (PMC8724785; doi:10.3389/fnagi.2021.756537)
Supplement: Supplementary file 1 [file Data_Sheet_1.docx]

**Supplementary Materials**

**Treadmill exercise during cerebral hypoperfusion has only limited effects on cognitive function in middle-aged subcortical ischemic vascular dementia mice**

Ryo Ohtomo^1, 2^, Hidehiro Ishikawa^1^, Keita Kinoshita^1,3^, Kelly K. Chung^1^, Gen Hamanaka^1^, Gaku Ohtomo^2^, Hajime Takase^1^, Christiane D. Wrann^4^, Hiroshi Katsuki^3^, Atsushi Iwata^5^, Josephine Lok^1^, Eng H. Lo^1^, Ken Arai^1^

1: Neuroprotection Research Laboratory, Departments of Radiology and Neurology, Massachusetts General Hospital and Harvard Medical School, Charlestown, MA 02129, USA

2: Department of Neurology, The University of Tokyo Graduate School of Medicine, Tokyo, Japan

3: Department of Chemico-Pharmacological Sciences, Graduate School of Pharmaceutical Sciences, Kumamoto University, Kumamoto 862-0973, Japan

4: Cardiovascular Research Center, Department of Medicine, Massachusetts General Hospital and Harvard Medical School, Charlestown, MA 02129, USA

5: Department of Neurology, Tokyo Metropolitan Geriatric Medical Center Hospital, Tokyo, Japan

Corresponding Author: Ken Arai,

Address: Neuroprotection Research Laboratory, MGH East 149-2401, Charlestown, MA 02129, USA.

e-mail: karai@partners.org

Contents:

- Supplementary methods

- Supplementary figures

**Supplementary Methods:**

**Laser-doppler flowmetry -** To evaluate the effect of BCAS surgery to the cerebral blood flow (CBF) over time, plastic guide cannula (outer diameter 3 mm, inner diameter 1.5mm, length 4 mm) was perpendicularly attached to the skull 2 mm posterior and 2 mm lateral to the bregma with dental cements (Sunmedical, Japan) after the removal of skin overlying the right skull of sham-operated and BCAS-operated mice. These procedures were conducted under anesthesia with isoflurane (4% for induction, 1.5% for maintenance). CBF was detected by inserting 1.5 mm probe into the cannula and analyzed with computer-based laser blood flowmeter (Perimed AB, Sweden). CBF was recorded shortly before and after sham/BCAS operation, and 1, 3, and 7 days after the surgery.

**Hematoxylin and Eosin staining (HE staining) -** To check whether cerebral perfusion by BCAS caused ischemic infraction in the cortex, coronal brain sections were processed for HE staining. The fresh frozen sections were fixed with 4% paraformaldehyde for 15 min, then rinsed with warm, running water for 1 min. After that, sections were stained with hematoxylin for 5 min to stain nuclei and rinsed for 3 min with water. Sections were then stained with eosin for 3 min, followed by rinsing for 1 min with water. Afterwards, the sections were dehydrated with 70% ethanol, 100% ethanol, and 100% ethanol (in respective order) for 10-15 seconds each. Following that, the sections were cleared using xylene.

**Supplementary Figures:**

**Supplementary Figure S1:** Laser-doppler flowmetry comparing CBF of BCAS mice and sham-operated mice. Data are expressed as mean ± SD, with 3 mice for each group. CBF dropped to ~50% of the original blood flow in BCAS mice immediately after the surgery, but gradually returned to ~70% by the 7th day of surgery.

**Supplementary Figure S2:** HE staining confirmed that cerebral hypoperfusion by BCAS did not cause a significant infarction in the cortex region in middle-aged mice.
